# A Population Genetic Perspective on Subsistence Systems in the Sahel/Savannah Belt of Africa and the Historical Role of Pastoralism

**DOI:** 10.3390/genes14030758

**Published:** 2023-03-20

**Authors:** Viktor Černý, Edita Priehodová, Cesar Fortes-Lima

**Affiliations:** 1 Archaeogenetics Laboratory, Institute of Archaeology of the Academy of Sciences of the Czech Republic, Letenská 1, 118 01 Prague, Czech Republic; 2 Human Evolution, Department of Organismal Biology, Evolutionary Biology Centre, Uppsala University, Norbyvägen 18C, 752 36 Uppsala, Sweden

**Keywords:** Africa, Sahel/Savannah belt, archaeogenetics, population genetics, selection, pastoralism

## Abstract

This review focuses on the Sahel/Savannah belt, a large region of Africa where two alternative subsistence systems (pastoralism and agriculture), nowadays, interact. It is a long-standing question whether the pastoralists became isolated here from other populations after cattle began to spread into Africa (~8 thousand years ago, kya) or, rather, began to merge with other populations, such as agropastoralists, after the domestication of sorghum and pearl millet (~5 kya) and with the subsequent spread of agriculture. If we look at lactase persistence, a trait closely associated with pastoral lifestyle, we see that its variants in current pastoralists distinguish them from their farmer neighbours. Most other (mostly neutral) genetic polymorphisms do not, however, indicate such clear differentiation between these groups; they suggest a common origin and/or an extensive gene flow. Genetic affinity and ecological symbiosis between the two subsistence systems can help us better understand the population history of this African region. In this review, we show that genomic datasets of modern Sahel/Savannah belt populations properly collected in local populations can complement the still insufficient archaeological research of this region, especially when dealing with the prehistory of mobile populations with perishable material culture and therefore precarious archaeological visibility.

## 1. Introduction

In the Sahel/Savannah belt of Africa (henceforth ‘the Sahel belt’), we can observe a mosaic of different ethnic groups, where various types of population interactions take place, including those between peoples of two distinct subsistence systems: nomadic pastoralists and sedentary farmers. With some simplification, one can say that farmers supply the market with grains and vegetables, while the pastoralists contribute meat and dairy products [1]. It should be noted that Sahelian nomadic pastoralists rely solely on natural pasturelands, meaning they do not grow forage for their animals. This clearly distinguishes this subsistence strategy from industrial farming, where animal husbandry and plant production tend to be more interconnected and geographically localised. In the Sahel, though, while the farmers are settled around their fields, build grain supplies in granaries, and live in permanent villages for generations, the pastoralists engage in transhumance and roam the countryside with their herds [2].

Ever since the colonial era, Sahelian pastoralists have been viewed as a source of instability and even blamed for the ecological crises caused by overgrazing. It was somewhat related to a theory called the ‘tragedy of the commons’ developed in the 1960s by the American environmentalist Garrett James Hardin [3]. According to it, if pasture is available to all herders with no restriction, everyone selfishly tries to increase the number of their animals until the pasture is exhausted. But a new concept of Sahelian pastoralism, which emerged in the late 1990s, is based on the observation that the Sahel is not a balanced ecosystem. Its vegetation cover is extremely unpredictable due to large local variations in the rainfall. Highly mobile pastoralism therefore cannot be harmful to the natural environment of the Sahel [4], because pastoralists do not stay in one place long enough to seriously damage the habitat.

**Figure 1:**
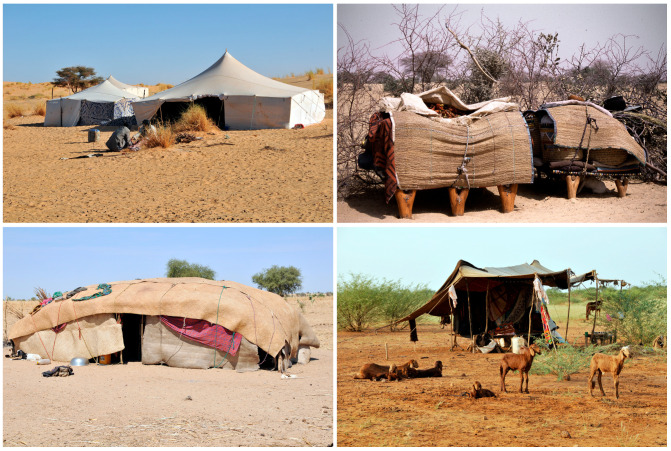
Examples of the camps and architecture of nomadic pastoralists in the Sahel belt. Top left: white fabric tents of the Moors in Mauritania; top right: tables for storing dishes at campsites of the Fulani Woɗaaɓe in Niger; bottom left: a tent made of mats in the camps of Baggara Arabs in Chad; and bottom right: lightweight and easily potable tents of the nomadic Shanablah Arabs in Kordofan, Sudan (Copyright: Viktor Černý).

Nowadays, from an ethnolinguistic point of view, we can distinguish between two main groups of pastoralists in the Sahel: the Fulani called Woɗaaɓe or Mbororo’en, who inhabit mainly the western part of the Sahel, mostly west of the Lake Chad or around it [5,6], and the Arabs called Baggara, Shuwa, or Kababish [7,8], who live in Sahel’s eastern part. Both of these populations herd mainly cattle. Further north, in the drier parts of southern Sahara, there are yet other groups of pastoralists who herd mostly camel. These include several populations, such as the Tubu divided to Daza and Teda [9,10], the Beri, also called Zaghawa or Bideyat [8,11], and many tribes of the Tuareg [12,13,14]. These nomadic peoples are geographically highly dispersed and stay at any particular site only for a relatively short time because of their transhumance lifestyle [15]. For this reason, they build easily transportable tents or camps (Figure 1), which show a large regional variation across the northern part of Africa [16].

Interestingly, pastoralism in the Sahel achieves an even higher level of protein production per hectare of land than livestock farming in climatically similar rainfall conditions abroad: while farmers in the US and Australia produce 0.3–0.5 kg of live weight per hectare of land, for Sahelian pastoralists practising traditional transhumance, it is 0.6–3.2 kg per hectare [17]. Moreover, some models even suggest that after the last wetter phase of the Holocene (~5.5 kya), pastoralism had slowed down the desertification of the Sahara and delayed the collapse of its ecosystem by at least 500 years [18]. It thus seems that pastoralism is beneficial for the semi-desert landscapes (drylands) in Africa and plays a highly important role in sustainable food-production strategies in the Sahel. A deeper understanding of Sahelian pastoralists based on population genetics is therefore a good starting point in endeavours to help reduce the risk of economic instability in this arid, remote, and unfortunately often overlooked part of Africa [19].

## 2. Archaeological Imprints of Pastoralism and Pastoralist’s Continuity

Skeletal remains found in southern Egypt, at the site of Nabta Playa and Bir Kiseiba in the western part of the Nile Valley, are considered to be the oldest evidence of the domestication of cattle in Africa [20,21]. Expeditions led by the Southern Methodist University (Dallas, TX, USA) in the 1980s have discovered almost 10,000 years old bones of cattle of a size smaller than would correspond to a non-domesticated individual, in this case an auroch [20,21]. The main argument pointing to cattle breeding, or rather care for semidomesticated animals, is based on the overall natural settings. According to researchers investigating the region on the border of Egypt and Sudan, animals that require regular access to natural water resources would not have been able to support themselves in this environment. The best explanation for their presence is therefore that they were taken there by humans.

**Figure 2:**
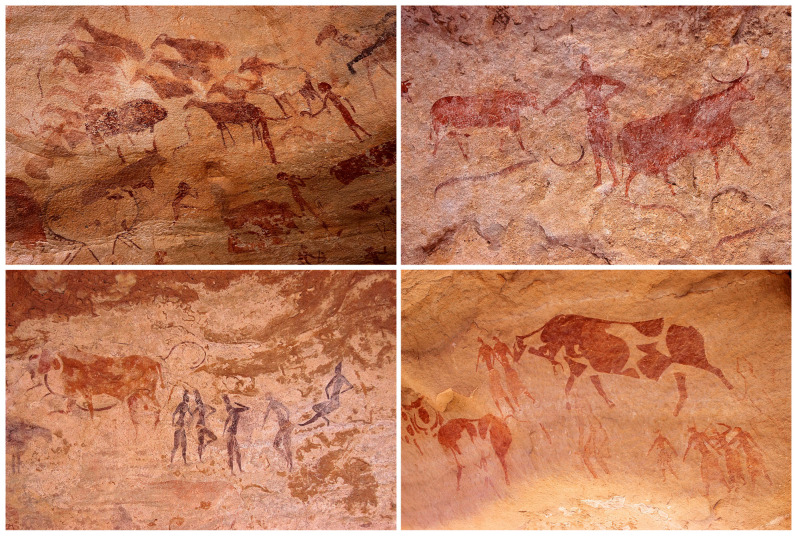
Rock art depicting pastoral scenes from Tassili n’Ajjer in Southern Algeria. Top left: sheep and goats in a row, probably tied with a rope; top right: figure of a herdsman with sub-Saharan anthropological features; bottom left: a pastoral scene painted in black and red (note that people in the middle have hairstyles reminiscent of the hair buns worn nowadays by the Fulani); bottom right: polychrome painting showing transhumance of a morphologically homogeneous herd of spotted cattle. Figures on the top are from the Sefar site, figures at the bottom from the Jabbaren site (Copyright: Viktor Černý).

African food-production strategies have been studied by various archaeological methods, often with the help of natural sciences [22]. It now seems highly probable that nomadic pastoralism preceded sedentary agriculture in Africa [23]. People in southern Libya milked their livestock as early as ~7 kya and moved around the vast areas of the Green Sahara [24]. Perhaps the best evidence of mobile pastoralism in Africa comes from Saharan rock art [25,26]. Figure 2 shows a few examples of rock paintings from southern Algeria documenting the so-called ‘pastoral period’. Although it is difficult to date these findings directly, the sequence in which the animals appear points to time estimates noted above [25,26]. 

Around 5.5 kya, however, traces of nomadic pastoralists gradually began to disappear. This disappearance was associated with a climate change that led to the desertification of the Sahara. Dating of archaeological sites in the northern part of Africa clearly shows that cattle spread from the northeast to the southwest over a relatively long period of time (~5000 years) [27]. In western Africa, similar to several sites in central and eastern Africa, nomadic pastoralists probably enriched their diet by hunting and gathering as well as occasional cultivation of pearl millet *Pennisetum glaucum*. Nevertheless, it is also possible that nomadic pastoralism was added to the cultural repertoire of west Africa only at the beginning of the Common Era [28]. Some archaeological investigations seem to indicate – based on the bones of cattle or other animals – the presence of animal husbandry, but it is hard to tell whether this is convincing evidence of nomadic pastoralism [29]. In fact, we must conclude that there is a gap in incontestable visibility of this specific subsistence strategy until the 19th century CE, when the pastoralists suddenly reappear in the written sources of the Masina Empire [30].

We can therefore pose the following three ‘taphonomic’ questions: Was pastoralism abandoned when the Green Sahara dried up around 5.5 kya? Was Sahelian pastoralism (re)invented only recently, in the 19th century, when it reappears in historical sources? Or is it rather so that its traces from the period between these time points simply do not survive? In fact, while farmers construct houses whose foundations remain archaeologically detectable for a long time, pastoralists build only simple mobile tents, which leave hardly any trace (see Figure 1). The issue of archaeological visibility of mobile peoples, such as the nomadic pastoralists, is difficult to address in African archaeology due to lack of archaeological evidence. Some archaeologists propose that one ought to combine ethno-archaeological fieldwork with geo-archaeological analyses [31], but this is practicable only on a small scale, in local settings, and not over large regions such as the Sahel belt.

## 3. Contributions of Population Genetics to the Understanding of Human Health

Since preservation of ancient DNA is in the Sahel environment with its high fluctuations in temperature and humidity highly unlikely, research tends to focus on the genetic diversity of modern populations, which is a hidden witness to their history. For instance, in genetic studies of microsatellite, mitochondrial DNA, and Y-chromosome data, the signature of some phylogenetic distances within pastoralist populations and the evidence of a gene flow between their local populations and the populations of farmers suggest that the groups may have coexisted in the past [32,33,34]. It implies that the abovementioned knowledge gap in the archaeological evidence is due to the absence of traces which pastoralists would leave—and not a sign of a (temporary) disappearance of pastoralism as a specific subsistence strategy.

When studying genetic adaptations, biological and cultural processes sometimes go hand in hand. An illustrative example is lactase persistence (LP), a trait genetically determined by several genetic variants, whose bearers can digest milk sugar even in adulthood. It was a considerable advantage for pastoralists with LP variants, as evidenced by the fact that it quickly began to spread in their populations through positive selection [35,36]. Across the Sahel, the frequency of individuals who can digest lactose from milk until adulthood is much higher among the pastoralists than among the farmers (see Table 2 in [37]). 

**Figure 3:**
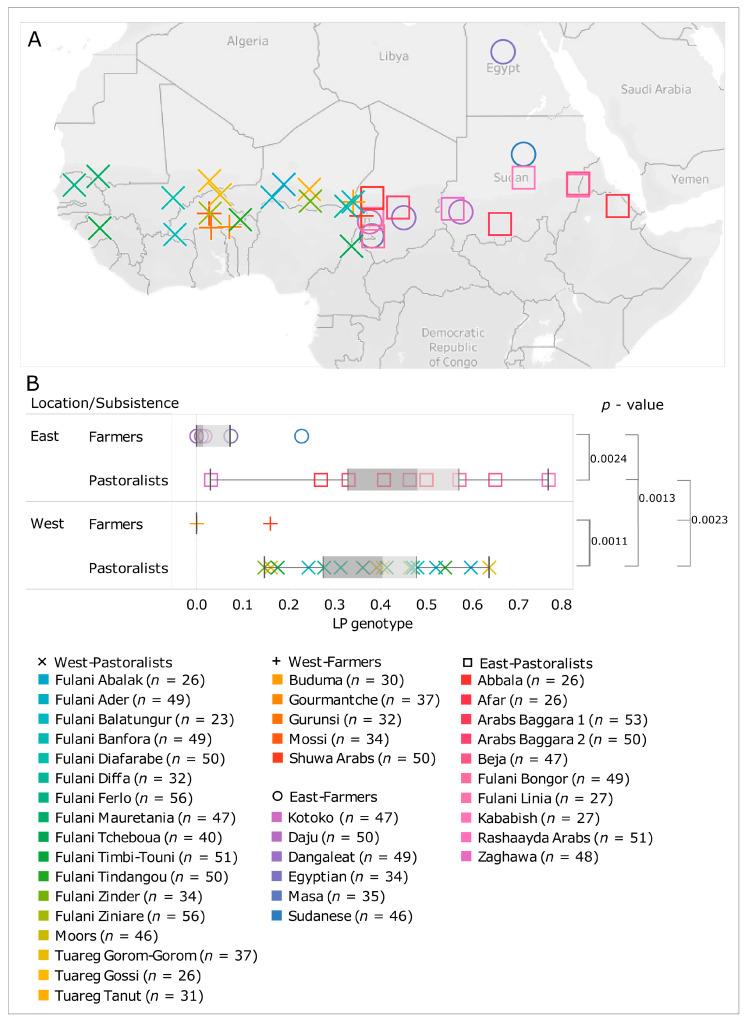
Lactase persistence (LP) genotype in the farmers and pastoralists from the Sahel belt. (**A**) Geographical locations of 38 pastoral and farmer populations analysed for LP mutations in previous studies [37,38,39]. (**B**) Box plot graphs of the analysed populations divided by the subsistence strategy (pastoralists and farmers) and by location with respect to Lake Chad (east and west). LP genotype is the sum of frequencies of the five LP variants (–13,910*T, –13,915*G, –14,010*C, –14,009*G, and –13,007*G) observed in each group. On the right, we report statistical significances (*p*-values) given by the Kruskal–Wallis test (test considered significant when *p* ≤ 0.05).

To illustrate this here, we have gathered the data of 38 populations analysed for LP in previous studies (Figure 3A) [37,38,39]. We split them in two groups (western and eastern) according to their position with respect to the Lake Chad, which was the crossroads of ancient migrations [34,37,40]. Figure 3B shows a clear difference between the distribution of five LP variants (–13,910*T, –13,915*G, –14,010*C, –14,009*G, and –13,007*G) in Sahelian populations grouped according to their lifestyle. The pastoralists from both the east and the west had a much higher occurrence of LP variants than their farmer neighbours (significant *p*-values estimated using the Kruskal–Wallis test confirmed this finding). The difference between eastern and western pastoralists was due to the different frequencies of five well-known genetic variants [37,38,39], which emerged and spread independently in different regions [36,41]. 

Western Sahelian pastoralists, especially the Fulani from Ziniaré in Burkina Faso, have LP variant –13,910*T, which is common in Europe [42]. It has been inferred by a method based on the linkage disequilibrium (LD) decay of haplotypes around this specific variant in this Fulani population that the ancestors of people with this variant must have encountered a population with some Eurasian ancestry. From this putative source, they acquired a haplotype with the –13,910*T variant via admixture no earlier than ~2 kya [42]. Further research has shown that this originally ‘European’ LP variant is found in the Sahel not only among the Fulani but also among many pastoralists with other linguistic affiliations, such as the Tuareg and Moors, but only in the western part of the Sahel belt [37]. This might suggest that the current pastoralists belong to a group that is older than expected. They may have ancient roots (older than ~2 kya) and further research is needed to better understand the origin and spread of nomadic pastoralism in the northern part of Africa.

A similar situation can be observed in the eastern Sahel, where we find a high frequency of a different LP variant (–13,915*G), previously described as an independent mutation associated with the domestication and milking of camels in the Arabian Peninsula ~4 kya [43]. This specific variant, also known as the Arabian LP variant, occurs mainly in Arabic-speaking populations [39] that migrated into the Sahel recently. It is found in many Arabic groups such as the Kababish, Baggara, or Shuwa, who all retained a nomadic lifestyle associated with the breeding of camels or other domestic animals. Based on estimates of its growth rate and the length of time of expansion [38], we can assume that this variant did not start to spread in the Sahel until ~1.4 kya, which neatly correlates with the beginning of historically documented expansion of Arabic tribes into Africa, and confirms the importance of population genetic research of current Sahelian populations and of Lake Chad as the border between the western and eastern part of the Sahel belt [40,44]. 

Another study dealing with genetic adaptations in the Sahel showed the importance of the *DARC* gene, which confers resistance against malaria caused by *Plasmodium vivax* [45]. While in the western part of the Sahel frequency of the protective allele is fixed and stable, in the eastern part of the Sahel its prevalence had increased via gene flow. Incidentally, there is one important fact perhaps related to this distribution: in the west of Africa, *P. vivax* is no longer present, and in the east it is declining, but other species of *Plasmodium* remain in the Sahel belt, such as *P. falciparum*. Malaria seems to have affected in a particular way also the genetic diversity of *HLA* genes of Sahelian populations, because the presence of alleles B*53:01:01 and B*78:01 closely correlates with environments where malaria is endemic [46]. These adaptations, which are not related to lifestyle but to the environment, were detected in both the pastoralists and the farmers. Still, it should be noted that the Fulani pastoralists are less clinically susceptible to malaria [47,48] because they develop a stronger inflammatory and antibody response against malaria parasites earlier on, even in early childhood [49]. Further research into transcriptional responses and regulations in several genes related to the immune system could shed further light on this subject [50].

## 4. The Contribution of Population Genetics to Disentangling Human History

Another potential target for exploring ancient gene flows between Sahelian groups of different subsistence strategies is the genetic diversity of uniparental systems. Both mitochondrial DNA (mtDNA) and nonrecombinant regions of the Y-chromosome (NRY) can exhibit sex-biased gene flow [51], as evidenced especially in recently admixed populations [52,53]. Asymmetric gene flow in uniparental markers has been detected in several Sahelian populations, especially among the Arab pastoralists who married local women of sub-Saharan origin [54].

Research into uniparental genetic systems across the Sahelian populations suggests that there might be an asymmetric gene flow between the pastoralists and the farmers on the level of mtDNA, i.e., the part of genome transmitted along the maternal line [55]. In the eastern part of the Sahel, this asymmetric gene flow has been stronger in the direction from the agricultural to the pastoralist populations, while in the western part of the Sahel it was less intense in that direction and clearly stronger in the opposite direction, i.e., from the pastoralists to the farmers. This indicates certain specific features of population dynamics within the populations of both subsistence strategies in the western and eastern Sahel, exemplified by the Fulani and the Arabic-speaking communities, respectively [55]. Another study [34] suggests, based on mtDNA and Y-chromosome data, that genetic distances among the local populations of farmers might significantly correlate with the geographic but not the linguistic distances. In the pastoralists, on the other hand, a significant correlation was found with the linguistic distances, but not with the genetic or the geographic ones, which is in agreement with their maternal and paternal genetic diversity [34]. Taken together, these findings indicate that genetic differentiation of sedentary farmers is determined by geography, while among the nomadic pastoralists, genetic differentiation correlates with language.

Joint analyses of uniparental genetic systems in conjunction with linguistics and geography might, therefore, help us better understand the sex-biased gene flow and, specifically, the asymmetrical migrations of men and women for both the pastoralists and the farmers. Further research could also shed light on the distribution of diversity of the X-chromosome. Unlike the autosomal chromosome, the X-chromosome has a sex-specific inheritance pattern, where females inherit two copies (one from the mother and one from the father) while males inherit only a single copy (from the mother). Although the X-chromosome plays a significant role especially in admixed populations, it is often neglected in population genetics [56,57,58]. In general, uniparental genetic systems indicate differences in the sex-specific contribution from the maternal or paternal lineage, differences in reproductive variance for males and females, and provide valuable information about gender differences in gene flow and human evolutionary history. Nevertheless, it is important to bear in mind that uniparental results are rather one-sided view and due to the relatively low effective size can distort our general view of the population history in Africa.

**Figure 4:**
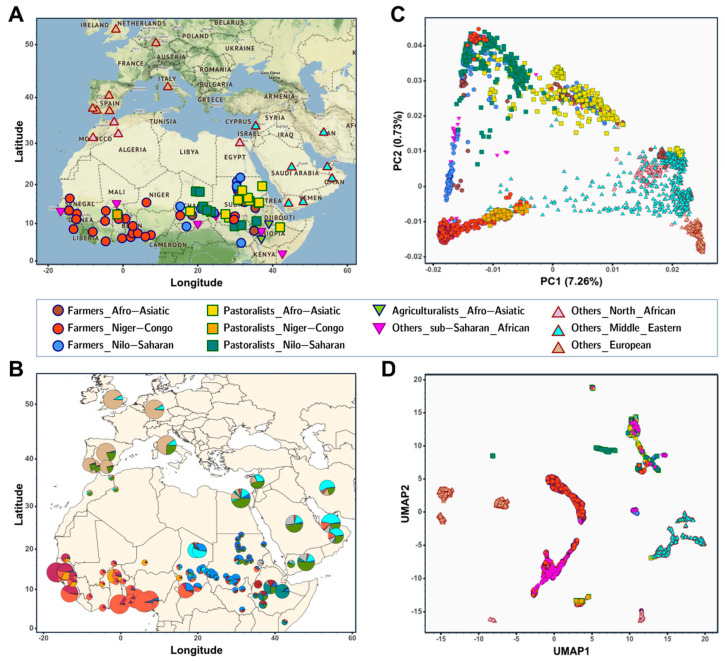
Patterns of admixture and population structure across the Sahel belt. (**A**) Geographic locations of populations included in the dataset (analysed were 3,245 individuals from 89 populations worldwide and 179,182 autosomal SNPs). This dataset was assembled after merging publicly available genome-wide SNP datasets [58,59,62,63,64,65,66,67] (further details have been presented previously; see supplementary Table S2 in [59]). (**B**) Genetic cluster membership estimated using unsupervised clustering ADMIXTURE analysis [60] at K = 12. The size of the pie charts is in relation to the sample size of each studied population and each colour shows the proportion of each estimated component. (**C**) Principal component analysis (PCA) for all studied populations estimated using the smartPCA [68]. (**D**) PCA-UMAP plot [61] combining the first five PCs estimated in the PCA. This figure highlights populations with different lifestyles (farmers by circles, pastoralists by squares, agriculturalists and other sub-Saharan African populations by inverted triangles, and other groups by triangles), speaking different African languages (Afro-Asiatic speakers in brown, yellow or green, Niger-Congo speakers in red or orange, and Nilo-Saharan speakers in blue or teal), or have different geographical distributions. For better visualisation, interactive figures were included and are available at https://github.com/cesarforteslima/Sahel-review, accessed on 20 January 2023.

Further insights into genetic diversity, admixture history, and population structure come from the analyses of genome-wide SNP data of African and West Eurasian populations (Figure 4A) [40,45,59]. Existing genome-wide studies show that it is rather difficult to distinguish between the abovementioned subsistence groups due to the complex patterns of genetic admixture between Sahelian populations with different historical and cultural backgrounds (Figure 4A) [59]. Unsupervised clustering methods [60] can suggest shared ancestry between populations which are geographically close (Figure 4B), differentiate between western and eastern Sahelian populations, and indicate likely patterns of population structure and gene flow in particular Sahelian populations with different continental ancestries, such as the putative patterns of gene flow in the Fulani and Arabic-speaker populations from Eurasian or sub-Saharan African sources, respectively [59].

Principal component analyses (PCA) can indicate population structure and distinguish between continental and subcontinental ancestries, such as sub-Saharan African populations and other populations with North African, Middle Eastern, or European ancestry in PC1, and between western and eastern African populations in PC2 (Figure 4C). To reveal a deeper population structure of the Sahelian populations, it is important to apply other dimensionality reduction methods, for example by combining PCA and the uniform manifold approximation and projection (UMAP) approach [61] to create PCA-UMAP plots (Figure 4D). Used together, clustering and dimensionality reduction methods provide new insights into the demographic history of Sahelian populations [59]. For instance, the genetic diversity of the pastoralist Fulani communities from the Sahel belt is a well-known example of complex admixture from diverse populations [42], where the high frequency of Eurasian ancestry in the Fulani groups due to admixture contributed to the spread of the European LP variant in their population [42,59].

Other differences between the pastoralists and the farmers in the Sahel belt can be demonstrated in the degree of inbreeding. The farmers tend to practice exogamy, while the pastoralists practice endogamy, which manifests itself among other things by a reduction of several genetic diversity measures in the mtDNA genome (such as gene diversity, nucleotide diversity, and the mean number of pairwise differences) of pastoralists analysed so far [69]. This finding is most evident in the Rashaayda Arab population from eastern Sudan, whose founders emigrated to the Sahel belt just ~150 years ago from Saudi Arabia [70]. This recent migrant pastoralist population is, therefore, rather exceptional in the Sahel belt due to extremely low or null levels of admixture with the sub-Saharan populations indicated by investigation of either their uniparental [34] or autosomal markers [59]. For instance, among all the Sahelian populations studied in [59], only the Rashaayda Arab population has similar values of the sum of short runs of homozygosity (ROH) (on average 348.6 ± 21.0 SD) as Middle Eastern populations (range: 326.4–350.9). These values differ from values observed in sub-Saharan African (range: 99.2–189.8), North African (range: 266.2–280.9), and European populations (range: 359.3–392.4). This population also has the highest values of the genomic inbreeding coefficient (F_ROH_ = 0.08 ± 0.03 SD) [59]. The Rashaayda Arab population is, therefore, in all the results, an outlier among African populations, both because it is more genetically similar to Middle Eastern populations and due to genetic drift, isolation, or inbreeding [59].

## 5. The Sociocultural Dynamics of Sahelian Populations

It is generally accepted that past demographic events—such as expansion, often accompanied by gene flow or admixture—have formed the genetic architecture of modern populations. Researchers who work with aDNA have direct material from historical populations but their research sometimes shows that these samples come from groups which did not substantially contributed their genetic heritage to present populations [71,72]. Consequently, adequate analyses of current local and geographically well-defined population groups can retroactively significantly contribute to our knowledge of prehistory of an African region [71]. This is especially true in the case of mobile and archaeologically almost invisible peoples, such as pastoralists, and/or in tropical and warm regions, such as the Sahel belt, where DNA preservation is often insufficient for successful aDNA analyses. 

One possible reason why traces of the middle Holocene Saharan pastoralists disappeared in the archaeological records could be linked to their secondary use of animals for transportation. It is possible that they established trans-Saharan trade networks. Copper, iron, and later also gold became the main drivers of their economy [73,74]. This list was soon expanded by the addition of salt [75,76], a crucial commodity for Sahelian farmers who in comparison with their hunter-gatherer ancestors gradually consumed more and more carbohydrates, and therefore needed salt to supplement their diet. Salt is still mined in the Sahara today: Well-known mines are, for example, the mines in Taudenni in northern Mali. From there, Tuareg pastoralists transport salt to Timbuktu, from where the salt slabs are transferred to ships sailing along the middle Niger Delta further south.

It is also interesting to note that, in contrast to other regions of the world, citadel-type buildings are not documented in the Sahel belt, although some places, such as Djenné in the inner Niger Delta, did sustain very large populations [77]. From this lack of evidence indicating settlement hierarchy some archaeologists deduced that pastoralists (or ‘mobile elites’) became not only the organisers of trans-Saharan trade but also built the first states in West Africa. Sahelian pastoralists probably traded various commodities mined outside the agricultural areas and over time created complex networks of local and long-distance exchange linking sub-Saharan Africa with the Mediterranean world [73]. As a result, the pastoralists may have used their animals not only for direct consumption or milking but also—and perhaps mainly—for transportation, a kind of use which can be included among items involved in the so-called secondary products revolution [78]. The introduction of camel, which originally provided transport between southern Arabia and the Mediterranean [79], later played a significant role also in the development of trans-Saharan trade. 

In this context, it is important to mention the so-called Baggarization, i.e., a trans-formation of Arab camel herders into cattle breeders, which took place after Arabic-speaking populations penetrated into the wetter environment east of the Lake Chad [80], where they came in contact with the Fulani pastoralists. In contrast, there is another process called Fulbeization, which refers to the assimilation of people from different, mostly small, ethnic groups into the Fulani society through conversion, interethnic marriage, or adoption [81]. This is related to social and ethnic identity, which undergoes changes in the course of interactions with different social groups [82]. These phenomena must be considered in population genetics as well, because local populations (demes) are currently labelled according to the ethnic groups. 

We should bear in mind that the social identity (lifestyle, language, ethnic, ...) of current populations in the Sahel does not necessarily indicate a shared cultural and genetic background. In this connection, let us recall the words of Eldridge Mohammadou, reflecting upon his own research in Cameroon: ‘*None of the ethnic components of this country can claim to have always been there: all, without exception, have at one time or another in the past originated outside the geopolitical triangle that is Cameroon today*’ [83]. A collaboration between geneticists, archaeologists, linguists, and ethnologists must, therefore, be based on a mutual confrontation and complementation of independent results.

Last but not least, we ought to emphasise that the maintenance of harmonic population interactions between pastoralists and farmers in the Sahel region is a most important but also highly complex matter. Fortunately, current disputes (over natural resources, especially water and land in climatically unfavourable years) are mostly resolved at the local level. Sometimes, though, they can escalate to a regional scale and even lead to humanitarian crises [84]. In such cases, it is good to realise which populations are the most vulnerable in the face of the upcoming climate crisis. The Intergovernmental Panel on Climate Change showed that the dry periods that occurred in the Sahel in the 1970s–1980s had a disproportionally greater negative impact on the pastoralists than on the farmers [85,86]. 

Unfortunately, current climate predictions are not optimistic. It is expected that by 2040, temperatures in the Sahel will increase on average by 2 °C and precipitation will decrease. According to some economists, this will lead to an increase in the market prices of cattle and consequently also other items [87]. There is, thus, a real risk that pastoralists might lose their traditional livelihood and engage in illegal trade with migrants [84]. It follows from the above that a deeper awareness of the historical relationships between the subsistence strategies across the Sahel belt can help us to better understand mutual population interactions between the pastoralists and the farmers and further research into the role of the pastoralists will help us form a more nuanced picture of human history of this region and of the African continent as a whole.

## 6. Conclusions

The genetic landscape of human populations across the Sahel belt has been shaped by past migrations, diverse linguistic groups, and complex social dynamics. Despite their complex demographic history, the genetic diversity of Sahelian populations is seldom explored in genomic research. Future research of underrepresented African populations from this region will thus improve our understanding of not only the populations of the Sahel belt but also of the patterns of genetic variation observed in Africa today. Socioeconomic and anthropological factors play an important role in shaping the admixture patterns and population structure observed in nomadic pastoralists and sedentary farmers. Due to this variety of relevant factors in populations with different subsistence systems, genetic results of the Sahelian populations should be interpreted with caution and always in conjunction with archaeological, linguistic, and historical sources. Such multidisciplinary studies of Sahelian populations can contribute to our knowledge of their population history and improve our understanding of human genetic diversity and health in Africa.

Last but not least, it should be noted that our research concerns only a relatively small part of the Sahelian metapopulation. There are many local groups which have not been properly sampled yet. In fact, numerous studies have shown that sub-Saharan Africa has the highest genetic diversity in the world, and almost every new research project that uses newly collected samples yields some unknown DNA sequences or haplotypes not present in current genomic datasets [88,89]. In this context, we should perhaps also mention research suggesting that some sub-Saharan African populations carry haplotypes that indicate introgression from yet unknown archaic hominins [90,91]. Similar to Eurasia, where the Neanderthals and Denisovans contributed to the genomic diversity in modern humans, also there may have been contacts between archaic and modern humans in sub-Saharan Africa (including the Sahel belt), which, due to environmental conditions, cannot be detected by related sciences, such as archaeology or palaeoanthropology. This is, thus, an area where population genetics could play a pioneering and irreplaceable role.

## Data Availability

No new data were created.
